# Swiss residents' arguments for and against a career in medicine

**DOI:** 10.1186/1472-6963-6-98

**Published:** 2006-08-14

**Authors:** Barbara Buddeberg-Fischer, Claudia Dietz, Richard Klaghofer, Claus Buddeberg

**Affiliations:** 1 Department of Psychosocial Medicine, Zurich University Hospital, Haldenbachstrasse 18, CH-8091 Zürich, Switzerland

## Abstract

**Background:**

In some Western countries, the medical profession is continuously losing prestige, doctors are claiming of high demands, low rewards, and difficult structural working conditions. This study aimed to investigate the arguments given by Swiss residents for and against a career in medicine.

**Methods:**

As part of a prospective cohort study of Swiss medical school graduates on career development, 567 fourth-year residents were asked to answer the free-response item of what arguments there still were in favour of or against a career in medicine. They also indicated whether they would choose the medical profession all over again (yes/no). The statements were transcribed, content categories inductively formulated, and their descriptions written down in a code manual. Arguments were encoded according to the code manual and assigned to eight content categories (Mayring's content analysis). Frequency distributions were given for categories and tested with Chi^2^-tests for differences in gender, speciality fields, and whether or not the respondent would again choose a career in medicine.

**Results:**

The 567 participants made 1,640 statements in favour of and 1,703 statements against a career in medicine. The content analysis of the residents' answers yielded eight categories with arguments both for and against a career in medicine. Of all "statements for" responses, 70% fell into the two top-ranking categories of *Personal experiences in day-to-day working life (41.2%) *and *Interpersonal experiences in professional relationships (28.8%)*. The top-ranking category of the "statements against" arguments was *General work-related structural conditions (32%)*, followed by *Social prestige and health-policy aspects (21%)*. Main arguments in favour of a career in medicine were interdisciplinary challenge, combination of basic sciences and interpersonal concerns, helping suffering people, guarantee of a secure job; arguments against comprised high workload, time pressure, emotional stress, poorly structured continuing education, increasing bureaucracy, work-life imbalance, low income, and decreasing social prestige. The statements revealed few differences depending on gender, medical field, and attitude towards choosing the medical profession again; one out of five young doctors would not do so.

**Conclusion:**

Residents' chief complaint is deteriorating structural working conditions, including unfavourable work-life balance. Making medicine an attractive profession again will require sustainable changes in health-policy framework and social reward.

## Background

As reported in several articles, physicians are becoming increasingly dissatisfied with many aspects of their professional life [1-6]. What reasons can be identified for the doctors' discontent and the considerable number of young physicians leaving the medical profession?

### Changing role of doctors in a changing society

Although physicians are spending more time with patients than in earlier periods, increased public and patient expectations towards the medical system and administrative and regulatory controls contribute to perceptions of increased time pressures and erosion of autonomy. As is known, these conditions lead to job strain [7].

### Work-life balance

Lifestyle and career orientation have changed in the younger generation, and this applies to doctors as well. Both male and female doctors are looking for a better balance between work and personal life. Not as many are willing to prioritize their professional career at the expense of their personal life [7-9].

### Residency programmes

Several studies report on the residents' dissatisfaction with their postgraduate training, both clinical and scientific [10,11]. Half of these residents complained of unstructured residency programmes – especially in surgical fields -leading to an extension of the time needed for qualifying in their speciality. Furthermore, a sizable number of doctors intending to specialize in surgery change their minds because of insufficient training and unfavourable workplace conditions.

### Income

One major reason for young physicians leaving medicine is the drop in income over the past decade [12]. Compared to other academics, return on educational investment over a working lifetime is fairly low [13]. Instead of embracing curative medicine and facing unattractive working conditions, they choose a career in health management, pharmaceutical companies, or medical informatics.

### Job satisfaction

Health care systems have a great impact on the working conditions and the doctors' job satisfaction [1,14-17]. State-administered health care systems seem to provide work environments which contribute to reasonably high job satisfaction as long as the professional autonomy is not eroded [15,17]. In competition-based settings, the job satisfaction declined in the last decades, mainly caused by financial incentives that strain doctors' professional principles, loss of control over their clinical decisions, and consuming administrative work.

### Career outside medicine

Some of the mentioned reasons might contribute that a considerable number of doctors prefer to pursue a career outside medicine. Nine years after graduating from medical school, 5% of the BMA cohort doctors had chosen a career outside curative medicine or had already left medicine [3]. In Germany, about one quarter of medical school graduates do not enter the medical profession [18]. Unfortunately, there are no such statistics for Switzerland.

As part of quantitatively assessed data of a prospective Swiss residents' career- development and life-satisfaction study [19,20] participants were asked (1) what arguments there still were in favour of or against a career in medicine, and (2) whether or not they would again choose to become doctors.

## Methods

### Study design

The present study is part of an ongoing prospective survey of a cohort of graduates of the three medical schools in German-speaking Switzerland, beginning in 2001 (T1). Of the 1,004 registered final-year students, 719 (72%) participated in the first assessment (T1) [19]. Subjects were re-evaluated after two years in 2003 (T2) [20,21]. The present paper refers to results of the third assessment (T3), conducted in the fourth year of residency (in 2005). The postal questionnaire consisted of multiple-choice and free-response items. The free-response items were embedded in the multiple-choice questions addressing the issues of what arguments still speak in favour of or against a career in medicine. A total of 577 subjects (306 females, 53%; 271 males, 47%) participated in the third assessment (T3).

The study was approved by the research and ethical committee of the Zurich University. The deans of the involved three medical faculties Basel, Bern and Zurich supported the survey by a letter of recommendation which was enclosed to the first inquiry. As the survey addressed healthy people on a voluntarily and anonymous basis without a planned intervention no further special ethical considerations had to be followed. To ensure participants' anonymity, the returned questionnaires were only identified by a code. The respondents sent their addresses to an independent address-administration office, allowing for follow-up.

### Instruments

The free-response items of the questionnaire read as follows: "*In your opinion, what arguments are there still in favour of or against a career in medicine? *("Welche Argumente sprechen Ihrer Meinung nach heute noch für bzw. gegen den Arztberuf?") *Please mention three arguments for and three against."*

In addition, participants answered the question of whether or not they would again choose to go into medicine ("yes" or "no") ("Würden Sie sich heute nochmals für den Arztberuf entscheiden?").

### Sample

Not all of the (n = 577) participants of the third assessment (T3) were included in the qualitative analysis. Ten of the respondents did not answer this question. The study sample therefore consisted of 567 residents (n = 303 females, 53.4%; n = 264 males, 46.6%). The mean age was 31.3 years (SD 2.4 y, range 27–46 y).

### Clinical fields and distribution of the residents

Residents were grouped according to the speciality qualification they aspired to. "Internal Medicine fields" comprised all sub-specialities of Internal Medicine and fields related to Internal Medicine, as well as Primary Care; Surgery, Obstetrics & Gynaecology, Urology, Orthopaedics, Ophthalmology and ENT were categorized as "Surgical fields"; "High-technology medicine" comprised Anaesthesiology, Emergency Medicine, Intensive Care, Nuclear Medicine, and Radiology. Further speciality groups were identified: "Paediatrics", "Psychiatry", "Other Specialities" such as Preventive Medicine, Pathology, and "Speciality not yet decided". Distribution of the aspired-to specialities of the 567 residents (100%) was as follows: Internal Medicine, n = 197 (34.7%), Surgical fields, n = 106 (18.7%), High-technology medicine, n = 51 (9.0%), Paediatrics, n = 48 (8.5%), Psychiatry, n = 27 (4.8%), Other specialities, n = 53 (9.3%), and Not yet decided, n = 85 (15.0%).

### Content and statistical analysis

The qualitatively assessed data (reasons for and against entering the medical profession) were evaluated according to Mayring's content analysis [22] by a researcher not involved in the quantitative data analysis, as follows: First, the respondents' handwritten answers (headwords or whole sentences) were transcribed into an Excel file. In a second step, *content categories *were inductively formulated, and their descriptions written down in a *code manual *(definition, examples, and rules for coding). In a further step, the passages of text were encoded according to the code manual and assigned to the content categories. Frequency distributions were given for categories and tested with Chi^2-^tests for differences in gender, speciality fields, and whether or not the respondent would again choose a career in medicine. *Inter-rater reliability*: A random sample of 20% of the analyzed statements was submitted to two additional raters (staff from the department experienced in qualitative analyses). The index of concordance (ratio of identically rated answers to all rated answers) was 0.79 and Cohen's Kappa 0.76.

## Results

### Categories of statements for and against a career in medicine

The 567 residents gave a total of 3,343 responses (5.90 per subject on average) in terms of both "statements for" and "statements against" a career in medicine. Of these, there were 1,640 "statements for" (mean 2.89, range 1 – 6) and 1,703 "statements against" (mean 3.00, range 1 – 6). I.e. some of the subjects gave more than three arguments, some less in terms of "statements for" and "statements against" a career in medicine respectively. The content analysis of the residents' answers resulted in eight categories with arguments both for and against a career in medicine. (The following order of the categories follows the ranking of the frequency distribution of the arguments given in favour of a career in medicine.)

- *Personal experiences in day-to-day working life*

- *Interpersonal experiences in professional relationships*

- *General work-related structural conditions*

- *Further training and speciality qualification conditions*

- *Enjoyment/Meaning*

- *Social prestige and health-policy aspects*

- *Income*

- *Leisure/Private life*

Non-distinctive statements such as "curiosity", "science", "the human being", "challenge" were assigned as *not codable*.

### Ranking and frequency distribution of statements for and against a career in medicine by gender

Table 1 shows the allocation of the responses to the eight categories differentiated according to "statements for" and "statements against" content by gender, and ranked by frequency of the "statements for" arguments.

Of all "statements for" responses of both men and women, 70% fell into the categories of *Personal experiences in day-to-day working life (41.2%) *and *Interpersonal experiences in professional relationships (28.8%)*, which occupied first and second place in the ranking for both genders. The top-ranking "statements against" responses were in the category *General work-related structural conditions *(32%), followed by *Social prestige and health-policy aspects *(21%). The frequency distributions of the eight categories and the "statements for" or "statements against" differ greatly according to their respective content.

The *ranking *of the *arguments for a career in medicine *categories was the same for male and female residents, while there were some differences in ranking between men and women in the *arguments against *categories: males rank negative arguments concerning *Income *in third place and *Leisure *towards the bottom, while females rank *Personal experiences in day-to-day working life *in third place and *Leisure/Private life *in fourth place.

#### Gender differences

Male participants gave significantly more "statements for" and "statements against" answers for the *Social prestige and health-policy *category than female residents, and also made significantly more critical statements vis-à-vis *Income*. Women named significantly more "statements against" answers for *Leisure/Private life *and *Personal experiences in day-to-day working life*.

Table 2 gives examples of residents' responses – both "statements for" and "statements against" a career in medicine – for each category (see also Additional file 1). The arguments for and against can be summarized as follows:

#### Main statements in favour of a career in medicine

The medical profession ...

- is an interdisciplinary challenge

- combines basic sciences and interpersonal concerns

- provides an opportunity to help suffering people

- covers a broad field of practice

- offers job security.

#### Main statements against a career in medicine

The medical profession ...

- is characterized by high workload, time pressure and emotional stress

- offers poorly structured speciality-qualification training

- is shifting away from work with patients to administrative work

- entails a work-life imbalance with limited quality of life

- is rewarded neither by adequate income nor by social acknowledgement.

### Frequency distribution of statements for and against a career in medicine by residents in different speciality fields

In a further step, we investigated whether the arguments for and against a career in medicine differed according to the residents' speciality field. As seen in Table 3.1 and 3.2, there were almost no differences between the statements of residents of the various medical fields, apart from significant differences for statements against a career in medicine in the category *Further training and speciality qualification conditions *for residents in High-technology medicine and Psychiatry; more negative entries were given in this category than for residents in the other speciality fields.

### Frequency distribution of statements for and against a career in medicine by residents who would choose to become a doctor all over again and those who would not

In addition, we were also interested in whether the frequency distribution of the given arguments for and against a career in medicine would vary according to whether or not residents would all over again decide to go into medical training (Table 4.1 and 4.2). Of the present study sample (n = 567), 442 (78%) participants (231 females, 76.2%; 211 males, 79.9%) would choose to become doctors again, while 117 (22%) residents (67 females, 22.1%; 50 males, 18.9%) would not. Five (1.7%) of the women doctors and 3 (1.1%) of the male doctors have not given a response. There were no significant gender-relevant differences.

As expected, residents who would not choose to go into medicine again named significantly more "statements against" in terms of *Personal experiences in day-to-day working life *(p < .05) and *Enjoyment/Meaning *(p < .05) (Table 4.2). They reported significantly fewer "statements for" in terms of *Enjoyment/Meaning *(p < .01) but surprisingly more in terms of *General work-related structural conditions *(p < .05) (Table 4.1).

## Discussion

In recent years, the image of medicine as a caring profession has been badly tarnished by a rash of critical reports in the media of many Western countries [5,23,24]. Blame for the rising costs of health care is laid mainly at the feet of doctors. To bring costs under control, managed-care bureaucrats increasingly limit the scope of doctors' work and patient care [4,17].

This *qualitative study *focused on the statements made by the participants of the prospective Swiss physicians' career-development and life-satisfaction study, in favour of or against a career in medicine. At the time of the assessment, the physicians were in the middle of their residency. The responses were evaluated according to Mayring's qualitative content analysis [22] and assigned to eight content categories.

Looking first at the *ranking and frequency distribution of residents' responses*, it is striking that the categories *Personal experiences in day-to-day working life *and *Interpersonal experiences *accounted for 70% of all **comments in favour of a career in medicine**. This indicates that most female and male physicians have chosen medicine because they have a high interest in the scientific and practical matters of the profession and are devoted to caring for people. Similar results are reported by McManus et al. [25]. Fourth-year residents seem to enjoy the practical experience of working as doctors with patients and value the interdisciplinary teamwork among the medical staff. In a previous assessment (T2), when the participants were in their first year of postgraduate training, statements concerning both positive and negative experiences of professional relationships were also ranked high [20]. The top-ranking **statements against a career in medicine **were the responses in the category *General work-related structural conditions *(32%). Heavy workload, on-call responsibilities and lack of mentorship and departmental support as well as increasing bureaucracy are disincentives to staying in clinical work [26]. Women doctors in particular complain that the current job-structure framework has a lasting negative effect on their career opportunities as soon as they attempt to balance their professional and personal lives [27]. Second place in the ranking of arguments against a career in medicine is taken by *Social prestige and health-policy aspects *(21%). To our knowledge, only a few studies have addressed these issues as deterring young doctors from remaining in curative medicine [5]. Accused by the media and health politicians of being greedy for power and money, doctors are often forced to legitimate their work on economic rather than scientific or ethical grounds. Because of increasing financial cutbacks and regulations passed by the government, doctors are restricted in their free professionalism and cannot properly plan their future basis of existence.

For *male residents *the latter reasons are even more important as arguments against a career in medicine than for *female residents*. Not surprisingly, there were few or almost no positive statements from either gender concerning *Income *and *Leisure/Private Life*. In terms of negative arguments in these categories, female residents made significantly fewer comments for *Income*, but more comments for *Private Life*. These assessments still reflect the gender stereotypes of men needing to provide financial support for their family while women identify more strongly with the caring aspect [28].

What makes the *medical profession still attractive *enough for young people so that a high number of school leavers apply for medical school? Study participants' statements, listed in Table 2 (see also Additional file 1), indicate that the bio-psychosocial profile of human medicine [29], the broad, interdisciplinary scope of academic medicine and the wide range of options for working as a doctor are key factors for the profession's appeal. A British study [25] also reported that the generic motivations of medical school applicants could be assigned to four dimensions: Indispensability, Helping People, Respect and Science. Unlike economics and fine arts, professional fields in which job security has decreased, the medical profession still provides a high degree of job security.

The *arguments against a career in medicine *mentioned above illustrate why men in particular have a declining interest in medicine: Badly structured residency programmes, heavy workload and inadequate wages are conditions which young physicians are no longer willing to accept. As seen in Great Britain [3] and Germany [18], an increasing number of medical-school graduates leave curative medicine, go abroad to countries with better working conditions [6], or express their dissatisfaction in demonstrations (Switzerland) or strikes (Germany).

Several decades ago, doctors tended to over-commitment to their jobs and neglect of their families. Nowadays, young physicians insist on a higher quality of life and more flexibility to arrange their personal lives according to their own tastes.

Contrary to the findings in the literature, we noted only a few differences in residents' statements depending on *different medical fields*. This result might be explained by the fact that unsatisfactory basic structural conditions of the profession are a major deterrent irrespective of the medical field. In surgical fields in particular, continuing education is often poorly structured [10,11,20,26], which leads to a declining interest in this field. In our study, High-technology medicine and Psychiatry residents gave significantly more arguments against a career in medicine in the *Quality of postgraduate training *category. It is probably safe to assume that the speciality-qualification programme guidelines are not properly implemented in these two medical fields.

That one in every five of the participating fourth-year residents would *not choose to become a doctor all over again *must be a cause for concern. Among the statements given against a career in medicine were a lack of enjoyment and fulfilment in patient care, and the experiencing of difficulties in day-to-day working life. At this point in their careers, it is unclear whether the aforementioned arguments against choosing a career in medicine will become so important that the residents leave medicine, or whether they will be able to cope with the unsatisfactory structural conditions of the medical profession and with the restrictions imposed by health policies. It is known that in other countries, especially Germany, a high percentage of doctors – more women than men – leave medicine after only the first year of clinical work [18]. Considering the high educational investments made in medical school graduates, in most European countries paid primarily by the government, better structural conditions in medicine must be established in order to retain the appeal of the profession.

Further assessments within the scope of the ongoing survey will contribute to answering the question of what careers the participating physicians will pursue in the long run.

## Conclusion

Residents complain mainly of deteriorating structural working conditions, including an unfavourable work-life balance. To make medicine attractive once again, sustainable changes in the conditions of the health-care system and social reward are essential.

## Competing interests

The author(s) declare that they have no competing interests.

## Authors' contributions

BBF, RK and CB designed the study, CD was responsible for data acquisition and conducted the content analysis, BBF, RK, CD and CB contributed to the interpretation of data, BBF drafted the manuscript versions, RK and CB revised the drafts critically for important intellectual content. All authors gave final approval to the version to be published.

## Pre-publication history

The pre-publication history for this paper can be accessed here:



## Supplementary Material

Additional File 1 Beispiele von Argumenten für oder gegen den Arztberuf. This Additional file gives the wording of the examples of the statements for and against a career in medicine in the original German language. Click here for file

## Figures and Tables

**Table 1:** Frequency distribution of "statements for" (n = 1,640 responses) and "statements against" (n = 1,703 responses) a career in medicine given by 264 male and 303 female residents, ranked by "statements for a career in medicine"

	**Statements for a career in medicine**			**Statements against a career in medicine**		
**Category**	**Males** **n (%)**	**Females** **n (%)**	**p**	**Males** **n (%)**	**Females** **n (%)**	**p**
Personal experiences in day-to-day working life	291 (38.7)	385 (43.4)	n.s.	80 (10.2)	124 (13.5)	< 0.05
Interpersonal experiences in professional relationships	208 (27.7)	264 (29.7)	n.s.	49 (6.2)	65 (7.1)	n.s.
General work-related structural conditions	79 (10.5)	73 (8.2)	n.s.	235 (29.9)	306 (33.3)	n.s.
Further training and speciality-qualification conditions	57 (7.6)	65 (7.3)	n.s.	64 (8.2)	75 (8.2)	n.s.
Enjoyment/Meaning	39 (5.2)	54 (6.1)	n.s.	3 (0.4)	7 (0.8)	n.s.
Social prestige and health-policy aspects	36 (4.8)	21 (2.4)	< 0.05	195 (24.8)	160 (17.4)	< 0.01
Income	13 (1.7)	7 (0.8)	n.s.	114 (14.5)	84 (9.2)	< 0.01
Leisure/Private life	0 (0)	1 (0.1)	1)	43 (5.5)	95 (10.3)	< 0.001
Not codable	29 (3.9)	18 (2.0)	0.03	2 (0.3)	2 (0.2)	n.s.
Total	752 (100)	888 (100)		785 (100)	918 (100)	

1) Computation of Chi^2 ^is not calculable because the total is too low (n = 1).

**Table 2:** Examples of residents' responses concerning "statements for" and "statements against" a career in medicine (see also Additional file 1)

**Category**	**Examples of 'statements for' a career in medicine**	**Examples of 'statements against' a career in medicine**
**Personal experiences in day-to-day working life**	"Professional, psychological and social challenge." "The combination of intellectual and manual work in surgery." "Working independently and with a high level of responsibility." "Variety in day-to-day work." "Medical issues offer a variety of solutions."	"High patient responsibility despite low clinical experience." "High workload under time pressure." "Emotional handling of difficult situations." "Health impairment caused by stress at work; afraid of experiencing burn-out." "A lot of routine, no space for acting autonomously."
**Interpersonal experiences in professional relationships **	"Working together with different teams and professionals." "Ability to help sick and suffering people." "Acknowledgement and gratefulness of patients." "The doctor-patient relationship is an interpersonal challenge."	"The patient is the king in hospital, hospitals are seen as 'shopping centres'." "Patients' overreaching expectations towards medicine and the medical professionals." "Little motivation and acknowledgement in the workplace." "Senior physicians' lack of communicative competence."
**General work-related structural conditions **	"The medical profession can be practiced all over the world, in different cultures, and under various social conditions." "There are still plenty of job opportunities, in hospital, private practice, management, and health policy."	"Old-fashioned hierarchical structures." "Heavy workload." "Shift from work with the patient to a lot of administrative work at the PC." "Difficult structural conditions for women who want to pursue a prestigious career."
**Further training and speciality qualification conditions**	"The medical profession gives one the opportunity of working in a broad field." "Interesting combination of basic sciences and interpersonal concerns."	"The speciality-qualification training is not at all well organized and structured." "Becoming a speciality trainee is often a matter of nepotism." "The fragmentation of the medical profession into many highly specialized disciplines makes doctors into narrow-minded nerds."
**Enjoyment/Meaning**	"Having the feeling that one is doing something 'good"'. "It's like a passion – one's career as a passion."	"You sacrifice yourself to the profession." "Modern medicine no longer sees human beings as whole people, but regards them as no more than disturbed functions or sick organs."
**Social prestige and health-policy aspects**	"Job security." "The medical profession still enjoys high social prestige."	"The medical profession is increasingly criticized by society." "Doctors are the scapegoats for rising costs in the health care system." "The health policy of the past few years has made the medical profession unattractive (Tarmed)." "Doctors constantly have to justify the treatment they administer to their patients to the health insurance companies." "The doctor-patient relationship has assumed an increasingly legal footing."
**Income**	"Secure income."	"Low income compared to other academics." "Considering the high workload, responsibility, and investment in education and training, the doctors' income is inadequately low."
**Leisure/Private life**	"Combination of work and family life with compromises in both areas."	"Tremendous cutback in social life." "Limited quality of life." "Difficulties for a woman in balancing job- and childcare-responsibilities"

**Table 3.1:** Frequency distribution of "statements for" a career in medicine (n = 1,640) given by residents of Internal medicine (n = 197), Surgical specialities (n = 106), High-technology medicine (n = 51), Paediatrics (n = 48), Psychiatry (n = 27), other specialities (n = 53), and residents not yet decided (n = 85)

**Statements for a career in medicine**								
**Category**	**Internal medicine** **n (%)**	**Surgical specialities** **n (%)**	**High-technology medicine** **n (%)**	**Paediatrics** **n (%)**	**Psychiatry** **n (%)**	**Other specialities** **n (%)**	**Not yet decided** **n (%)**	**p**
Personal experiences in day-to-day working life	242 (42.1)	126 (41.9)	63 (42.6)	56 (40.3)	34 (43.0)	55 (35.7)	100 (41.0)	n.s.
Interpersonal experiences in professional relationships	171 (29.7)	87 (28.9)	34 (23.0)	47 (33.8)	26 (32.9)	41 (26.6)	51 (20.9)	n.s.
General work-related structural conditions	53 (9.2)	20 (6.6)	13 (8.8)	9 (6.5)	6 (7.6)	21 (13.6)	30 (12.3)	n.s.
Further training and speciality qualification conditions	35 (6.1)	19 (6.3)	14 (9.5)	10 (7.2)	4 (5.1)	14 (9.1)	26 (10.7)	n.s.
Enjoyment/Meaning	27 (4.7)	23 (7.6)	9 (6.1)	11 (7.9)	3 (3.8)	11 (7.1)	9 (3.7)	n.s.
Social prestige and health-policy aspects	20 (3.5)	14 (4.7)	5 (3.4)	1 (0.7)	3 (3.8)	6 (3.9)	8 (3.3)	n.s.
Income	9 (1.6)	3 (1.0)	2 (1.4)	0 (0)	2 (2.5)	1 (0.6)	3 (1.2)	1)
Leisure/Private life	0 (0)	0 (0)	0 (0)	1 (0.7)	0 (0)	0 (0)	15 (6.1)	1)
Not codable	18 (3.1)	9 (3.0)	8 (5.4)	4 (2.9)	1 (1.3)	5 (3.2)	2 (0.8)	n.s.
Total	575 (100.0)	301 (100.0)	148 (100.0)	139 (100.0)	79 (100.0)	154 (100.0)	244 (100.0)	

1) Computation of Chi^2 ^is not calculable because the total is too low (n = 1)

**Table 3.2:** Frequency distribution of "statements against" a career in medicine (n = 1,703) given by residents of Internal medicine (n = 197), Surgical specialities (n = 106), High-technology medicine (n = 51), Paediatrics (n = 48), Psychiatry (n = 27), other specialities (n = 53), and residents not yet decided (n = 85)

**Statements against a career in medicine**								
**Category**	**Internal medicine** **n (%)**	**Surgical specialities** **n (%)**	**High-technology medicine** **n (%)**	**Paediatrics** **n (%)**	**Psychiatry** **n (%)**	**Other specialities** **n (%)**	**Not yet decided** **n (%)**	**p**
Personal experiences in day-to-day working life	83 (14.3)	29 (9.0)	17 (11.3)	15 (10.4)	10 (12.7)	15 (9.2)	35 (13.3)	n.s.
Interpersonal experiences in professional relationships	32 (5.5)	24 (7.4)	11 (7.3)	16 (11.1)	4 (5.1)	12 (7.4)	15 (5.7)	n.s.
General work-related structural conditions	182 (31.4)	109 (33.6)	38 (25.3)	51 (35.4)	21 (26.6)	48 (29.4)	92 (35.0)	n.s.
Further training and speciality qualification conditions	36 (6.2)	32 (9.9)	18 (12.0)	6 (4.2)	14 (17.7)	13 (8.0)	20 (7.6)	0.01
Enjoyment/Meaning	3 (0.5)	1 (0.3)	1 (0.7)	1 (0.7)	0 (0)	1 (0.6)	3 (1.1)	1)
Social prestige and health-policy aspects	130 (22.4)	59 (18.2)	29 (19.3)	22 (15.3)	17 (21.5)	43 (26.4)	55 (20.9)	n.s.
Income	63 (10.9)	47 (14.5)	16 (10.7)	20 (13.9)	9 (11.4)	24 (14.7)	19 (7.2)	n.s.
Leisure/Private life	50 (8.6)	22 (6.8)	20 (13.3)	13 (9.0)	3 (3.8)	7 (4.3)	23 (8.7)	n.s.
Not codable	1 (0.2)	1 (0.3)	0 (0)	0 (0)	1 (1.3)	0 (0)	1 (0.4)	n.s.
Total	580 (100.0)	324 (100.0)	150 (100.0)	144 (100.0)	79 (100.0)	163 (100.0)	263 (100.0)	

1) Computation of Chi^2 ^is not calculable because the total is too low (n = 1)

**Table 4.1:** Frequency distribution of "statements for" a career in medicine (n = 1,640 responses) given by 442 residents who would go into medicine again and 117 residents who would not, as well as by 8 residents not giving a response.

	**Statements for a career in medicine**			
**Category**	**Statements of residents who would go into medicine again** **n (%)**	**Statements of residents who would not go into medicine again** **n (%)**	**No response in terms of choosing medicine again or not** **n (%)**	**p**
Personal experiences in day-to-day working life	536 (41.4)	128 (39.5)	12 (54.5)	n.s.
Interpersonal experiences in professional relationships	372 (28.7)	95 (29.3)	5 (22.7)	n.s.
General work-related structural conditions	106 (8.2)	44 (13.6)	2 (9.1)	<0.05
Further training and speciality qualification conditions	99 (7.7)	22 (6.8)	1 (4.5)	n.s.
Enjoyment/Meaning	83 (6.4)	9 (2.8)	1 (4.5)	<0.01
Social prestige and health- policy aspects	50 (3.9)	6 (1.9)	1 (4.5)	n.s.
Income	14 (1.1)	6 (1.9)	0 (0)	n.s.
Leisure/Private life	1 (0.1)	0 (0)	0 (0)	1)
Not codable	33 (2.6)	14 (4.3)	0 (0)	n.s.
Total	1294 (100.0)	324 (100.0)	22 (100.0)	

1) Computation of Chi^2 ^is not calculable because the total is too low (n = 1)

**Table 4.2:** Frequency distribution of "statements against" a career in medicine (n = 1,703 responses) given by 442 residents who would go into medicine again and 117 residents who would not, as well as by 8 residents not giving a response.

	**Statements against a career in medicine**			
**Category**	**Statements of residents who would go into medicine again** **n (%)**	**Statements of residents who would not go into medicine again** **n (%)**	**No response in terms of choosing medicine again or not** **n (%)**	**p**
Personal experiences in day-to-day working life	143 (11.0)	52 (13.8)	9 (39.1)	<0.05
Interpersonal experiences in professional relationships	84 (6.5)	29 (7.7)	1 (4.3)	n.s.
General work-related structural conditions	418 (32.1)	119 (31.5)	4 (17.4)	n.s.
Further training and speciality qualification conditions	104 (8.0)	34 (9.0)	1 (4.3)	n.s.
Enjoyment/Meaning	4 (0.3)	5 (1.3)	1 (4.3)	<0.05
Social prestige and health- policy aspects	285 (21.9)	68 (18.0)	2 (8.7)	n.s.
Income	153 (11.8)	42 (11.1)	3 (13.0)	n.s.
Leisure/Private life	109 (8.4)	27 (7.1)	2 (8.7)	n.s.
Not codable	2 (0.2)	2 (0.5)	0 (0)	n.s.
Total	1302 (100.0)	378 (100.0)	23 (100.0)	

1) Computation of Chi^2 ^is not calculable because the total is too low (n = 1)
