# Supplementary material for: Swiss residents' arguments for and against a career in medicine
Source: BMC Health Serv Res. 2006 Aug 14;6:98. doi: 10.1186/1472-6963-6-98 (PMC1564007; doi:10.1186/1472-6963-6-98)
Supplement: Additional File 1 — Beispiele von Argumenten für oder gegen den Arztberuf. This Additional file gives the wording of the examples of the statements for and against a career in medicine in the original German language. [file 1472-6963-6-98-S1.doc]

# Additional files

Additional file 1 – Beispiele von Argumenten für oder gegen den Arztberuf

| **Kategorie** | **Beispiele von Argumenten für den Arztberuf** | **Beispiele von Argumenten gegen den Arztberuf** |
| --- | --- | --- |
| **Persönliche Erfahrungen im Berufsalltag** | „Fachliche, psychologische und soziale Herausforderung.”  „Kombination von intellektueller und manueller Tätigkeit in der Chirurgie.”  „Selbständiges Arbeiten mit einem hohen Mass an Verantwortung.”  „Abwechslungsreiche Tätigkeit.”  „Medizinische Fragestellungen bieten viele Lösungsmöglichkeiten.” | „Grosse Verantwortung für Patienten trotz geringer klinischer Erfahrung.”  „Hohe Arbeitsbelastung unter Zeitdruck.”  „Emotionale Belastung durch schwierige Situationen.”  „Gesundheitliche Beeinträchtigung durch Stress bei der Arbeit, Angst vor Burn-out.”  „Viel Routine, wenig Raum für eigenständiges Handeln.” |
| Beziehungserfahrun-gen im Berufsalltag | „Zusammenarbeit mit verschiedenen Teams und Berufsgruppen.”  „Möglichkeit, kranken Menschen zu helfen.”  „Anerkennung und Dankbarkeit von Patienten.”  „Die Arzt-Patient-Beziehung ist eine zwischenmenschliche Herausforderung.” | „Der Patient ist König im Spital, Spitäler werden oft als ‘shopping- centers’ betrachtet.  „Übersteigerte Ansprüche der Patienten an die Medizin und die medizinischen Fachpersonen.”  „Wenig Anerkennung und Motivierung am Arbeitsplatz durch Vorgesetzte.”  „Mangelnde soziale und kommunikative Kompetenz der Vorgesetzten.” |
| **Strukturelle berufliche Rahmenbedingungen** | “Der Arztberuf kann auf der ganzen Welt, in verschiedenen Kulturen und unter verschiedenen sozialen Bedingungen ausgeübt werden.”  „Es gibt immer noch viele Berufsmöglichkeiten, z.B. im Spital, in der Privatpraxis, im Management oder in der Gesundheitspolitik.” | „Überholte hierarchische Strukturen.”  „Grosse Arbeitsbelastung.”  „Verschiebung der Arbeit mit dem Patienten zu immer mehr administrativer Arbeit am PC, Bürokratisierung des Arztberufs.”  „Schwierige berufliche Rahmenbedingungen für Frauen, die neben der Familie noch eine anspruchsvolle Karriere verfolgen möchten.” |
| Aus-, Weiter- und Fortbildung | “Der Arztberuf bietet ein breites Betätigungsfeld mit vielen Weiter- und Fortbildungsmöglichkeiten.”  „Interessante Mischung von Naturwissenschaften und zwischenmenschlichen Belangen.” | „Die fachärztliche Weiterbildung ist nicht gut organisiert und strukturiert.”  „Eine Weiterbildungsstelle zu bekommen, ist oft eine Frage von ‚Vetternwirtschaft’.”  „Die Fragmentierung der Medizin in viele hoch spezialisierte Fachdisziplinen macht Ärzte zu ‚Fachidioten’.” |
| **Freude / Sinn** | “Als Arzt hat man das Gefühl, etwas ‚Gutes’ auf der Welt zu tun”.  „Es ist wie eine Leidenschaft – der Beruf ist eine Passion.” | „Man opfert sich für den Beruf auf.”  „Die moderne Medizin betrachtet die Patienten nicht mehr als ganze Menschen, sondern sieht nur noch gestörte Funktionen oder kranke Organe.” |
| **Sozialprestige und gesundheitspolitische Aspekte** | „Sicherer Arbeitsplatz.”  „Der Arztberuf hat immer noch ein hohes Sozialprestige.” | „Die Ärzte werden zunehmend von der Gesellschaft kritisiert.”  „Ärzte sind die Sündenböcke für die steigenden Kosten im Gesundheitswesen.”  „Die Gesundheitspolitik der letzten Jahre machte den Arztberuf unattraktiv (z.B. Tarmed).”  „Ärzte müssen ihre Behandlungsmassnahmen gegenüber den Krankenkassen ständig rechtfertigen.”  „Die Arzt-Patient-Beziehung wird zunehmend ‚verrechtlicht’.” |
| **Einkommen** | „Sicheres Einkommen.” | „Niedriges Einkommen im Vergleich mit anderen akademischen Berufen.”  „Betrachtet man die hohe Arbeitsbelastung, Verantwortung und die Aus- und Weiterbildungskosten, ist das Einkommen der Ärzte inadäquat niedrig.” |
| **Freizeit / Privatleben** | „Beruf und Familie sind mit Kompromissen auf beiden Seiten immer besser vereinbar.” | „Starke Einschränkung des Soziallebens.”  „Geringe Lebensqualität.”  „Schwierigkeiten für eine Frau, Beruf und Familie unter einen Hut zu bringen.” |
